# Effect of concomitant use of memantine on mortality and efficacy outcomes of galantamine-treated patients with Alzheimer’s disease: post-hoc analysis of a randomized placebo-controlled study

**DOI:** 10.1186/s13195-016-0214-x

**Published:** 2016-11-15

**Authors:** Klaus Hager, Alan S. Baseman, Jeffrey S. Nye, H. Robert Brashear, John Han, Mary Sano, Bonnie Davis, Henry M. Richards

**Affiliations:** 1 Clinic for Medicine of the Elderly, Hannover, Germany; 2 Janssen Research and Development, LLC, Titusville, NJ USA; 3 The Mount Sinai Medical Center, New York, NY USA; 4 Synaptec Inc, Palm Beach Gardens, FL USA; 5 Janssen Research and Development, LLC, 850 Ridgeview Drive, Horsham, PA 19044 USA

**Keywords:** Baseline scores, Galantamine, Memantine, Mortality, Alzheimer’s disease

## Abstract

**Background:**

A large, prospective, 2-year, randomized study in patients with mild-to-moderate Alzheimer’s disease or mixed dementia demonstrated reductions in mortality and cognitive/functional decline in galantamine-treated patients. A post-hoc analysis was conducted to study the effect of (the presence or absence of) concomitant memantine use on treatment outcome.

**Methods:**

Randomized patients (*N* = 2045) were divided into subgroups based on memantine use. Analyses included demographic and clinical characteristics (age, nursing home placement, Mini-Mental State Examination (MMSE) and Disability Assessment for Dementia (DAD) scores) and mortality endpoints.

**Results:**

Overall, 496 (24.3 %) patients were memantine users and were older (mean (SD), 74.0 (8.76) vs 72.8 (8.76), *p* = 0.008), with lower MMSE scores (18.2 (4.16) vs 19.2 (4.02), *p* < 0.0001) and DAD scores (58.0 (23.49) vs 62.5 (20.52), *p* < 0.0001) than nonusers. Mortality rates (per 100 patient-years) in memantine nonusers (*n* = 1549) were lower for galantamine (1.39) vs placebo-treated patients (4.15). In memantine users, mortality rates were similar for placebo-treated (4.49) and galantamine-treated patients (5.57). In memantine nonusers at 24 months, the decline in MMSE scores (effect size (95 % CI) 0.25 (0.14; 0.36)) and DAD scores (0.17 (0.06; 0.28)) from baseline was lower in galantamine patients vs placebo patients. The absence of these benefits in memantine users could not be explained by baseline age, MMSE, or DAD scores.

**Conclusion:**

This post-hoc analysis shows that the beneficial effects of galantamine at 2 years post treatment were not observed in patients who had been placed on background memantine. The reasons for memantine treatment and the possibility of interaction between memantine and galantamine merit further investigation.

**Trial registration:**

ClinicalTrials.gov NCT00679627. Registered 15 May 2008.

Electronic supplementary material

The online version of this article (doi:10.1186/s13195-016-0214-x) contains supplementary material, which is available to authorized users.

## Background

Cholinesterase inhibitors have symptomatic benefits across a wide range of dementia severity, as demonstrated in short-term controlled studies ranging from 6 months to 1 year. In addition to cognitive, functional, and behavioral benefits, the first report of these studies demonstrated that galantamine also reduced mortality and demonstrated cognitive benefit for up to 2 years [1].

Galantamine HBr is a reversible, competitive cholinesterase inhibitor and a positive allosteric modulator of nicotinic receptors [2] approved in the USA for the treatment of mild-to-moderately severe dementia of Alzheimer type, and for Alzheimer’s disease (AD) with cerebrovascular disease in certain other countries. The efficacy and safety of galantamine have been demonstrated in several phase III, double-blind, randomized, controlled trials [1, 3–6]. Memantine, a noncompetitive N-methyl-D-aspartate (NMDA) receptor antagonist and open-channel blocker of nicotinic receptors, is approved in the USA for the treatment of patients with moderate-to-severe dementia of AD [7]. Memantine may be used in combination with a cholinesterase inhibitor because the two have different mechanisms of action and the combination has been thought to further improve cognitive processing [7, 8].

A randomized, placebo-controlled study [1] conducted to evaluate the long-term (2-year) efficacy and safety of galantamine in patients with mild-to-moderate AD showed beneficial effects of galantamine on mortality, as well as cognitive and functional decline. The present post-hoc analysis of that study was conducted to evaluate the effect of memantine on treatment outcome as patients who were already taking stable doses of memantine were allowed to continue the treatment.

## Methods

The primary study was a randomized, double-blind, placebo-controlled, parallel-group, multicenter study of galantamine in patients with mild-to-moderately severe AD, with and without cerebrovascular disease [1]. The study was conducted from May 2008 to May 2012 at 127 centers in European countries. Details of the study design have been reported previously [1]. A total of 2051 patients were randomized (1:1) to galantamine or placebo treatment for up to 24 months. Memantine hydrochloride was taken at baseline and throughout the study by 24.5 % of galantamine-treated patients and 24.0 % of placebo-treated patients. Nursing home placement was queried with the following question from the informant: “During the past 3 months, did your relative/friend reside at home or in an establishment providing supported accommodation?” The respondent was asked to choose among: own home (alone), own home (with relative/friend), supported/sheltered housing, residential home and nursing home, and to indicate whether temporary or permanent.

This post-hoc analysis of the primary study was conducted to evaluate the effect of memantine on mortality and efficacy parameters including Mini-Mental State Examination (MMSE) scores, Disability Assessment for Dementia (DAD) scores, and nursing home placement of galantamine-treated patients with AD and mixed dementia.

Patients with these variables were divided into subgroups by the use and nonuse of memantine: median age at baseline (<median; ≥median), MMSE score (10–17; 18–26), and DAD score (<median; ≥median). The effect of memantine use was determined on mortality, MMSE, and DAD endpoints in these subgroups.

### Additional analyses

Prior medical history, concomitant medications, treatment emergent adverse events (TEAEs), TEAEs leading to death, deaths, and nursing home placement were summarized by the use and nonuse of memantine.

### Statistical methods

Baseline variables and nursing home placement were compared by *t* test or chi-square test as appropriate. Effect sizes and mean differences were calculated for MMSE and DAD values. Other analyses were summarized descriptively. Mortality rate and change from baseline in MMSE and DAD scores at month 24 (last observation carried forward (LOCF) analysis) were summarized by baseline characteristics. Hazard ratio (HR) and 95 % CI (mortality) and the mean difference and 95 % CI (MMSE and DAD) for different treatment groups were displayed using forest plots for each subgroup. Intention to treat (ITT) with the LOCF approach was used for MMSE and DAD analysis. The ITT analysis set included all randomized patients who had at least one post-baseline MMSE measure. Mortality analyses were based on the data from the safety analysis set, which included all randomized and treated patients. In addition, other baseline characteristics were summarized descriptively to consider other possible effects.

## Results

### Study participants

This post-hoc analysis included 2045 patients with mild-to-moderate AD or mixed dementia. The majority of patients were women and white. Baseline characteristics of the patient subgroups divided by memantine use or nonuse, and randomization to placebo or galantamine, are presented in Table 1. Memantine users had slightly lower MMSE and DAD scores compared with the nonusers (MMSE: 18.2 vs 19.2, *p* < 0.0001; DAD: 58.0 vs 62.5, *p* < 0.0001). The duration of AD was similar, but 23.4 % of memantine users, compared with 15.2 % of nonusers, had a history of cholinomimetic treatment (*p* < 0.0001). Nursing home residence was recorded at baseline for 6.0 % of memantine users and 4.4 % of nonusers (*p* = 0.35). Medical history at baseline was notable for differences in cardiovascular disease (memantine users: 72.1 %; memantine nonusers: 65 %), neurologic disease (memantine users: 33.1 %, memantine nonusers: 24.7 %), and psychiatric disease (memantine users: 22.8 %; memantine nonusers: 16.6 %) (Additional file 1: Table S1). The medications taken at baseline were consistent with the medical history of patients (Additional file 2: Table S2).

**Table 1:** Baseline demographics of patients with and without concomitant memantine use

	Memantine		No memantine		All		
	Placebo	Galantamine	Placebo	Galantamine	Memantine	No memantine	*p* value
*N*	245	251	776	773	496	1549	
Sex							
Women (%)	60.4	67.3	65.2	64.9	63.9	65.1	0.7976^χ^
Age (years)							
Mean (SD)	74.2 (8.7)	73.8 (8.8)	72.8 (8.6)	72.7 (8.9)	74.0 (8.76)	72.8 (8.76)	0.008^t^
MMSE							
Mean (SD)	18.4 (4.2)	18.0 (4.1)	19.1 (4.0)	19.4 (4.1)	18.2 (4.16)	19.2 (4.02)	0.0001^t^
DAD							
Mean (SD)	59.0 (23.3)	57.0 (23.7)	61.5 (20.3)	63.4 (20.7)	58.0 (23.49)	62.5 (20.52)	0.0001^t^
AD duration (years)							
Mean (SD)	0.8 (1.3)	1.1 (1.7)	0.8 (1.7)	0.7 (1.3)	0.95 (1.50)	0.75 (1.57)	0.0127^t^
Prior therapy							
Cholinomimetic use (%)	23.3	23.5	16.0	13.3	23.4	14.7	0.0001^χ^
Nursing home placement							
*N*	116	101	312	305	217	617	
Cases, *n* (%)	10 (8.6)	3 (3.0)	14 (4.5)	13 (4.3)	13 (6.0)	27 (4.4)	0.3507^χ^

^*χ*^Chi-square analysis

^*t*^ *t* test

*AD* Alzheimer’s disease, *DAD* disability assessment for dementia, *MMSE* mini–mental state examination, *SD* standard deviation

### Outcome: mortality

**Fig. 1:**
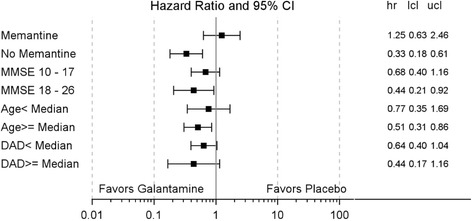
Time to death by subgroup. *DAD* Disability Assessment for Dementia, *MMSE* Mini-Mental State Examination, *hr* hazard ratio, *lcl* lower confidence limit, *ucl* upper confidence limit

In memantine users, mortality rates were not reduced by galantamine (HR: 1.25; 95 % CI: 0.63; 2.46) as they were in nonusers (HR: 0.33; 95 % CI: 0.18; 0.61) (Table 2). Mortality rates in the galantamine-treated groups, compared with placebo, were lower in patient groups with ≥ median age and higher MMSE score (18–26) (Additional file 3: Table S3). Results for the other subgroups generally showed a trend in favor of galantamine, regardless of age, MMSE values, or DAD values at baseline (Fig. 1). The reduction in mortality observed with galantamine treatment of memantine nonusers was distributed across organ systems (Additional file 4: Table S4).

**Table 2:** Mortality, cognitive, functional, and disposition outcome measures

	Memantine		No memantine		Placebo
	Placebo	Galantamine	Placebo	Galantamine	Memantine vs no memantine
Time to death					
*n*/*J* (%)	15/245 (6.1)	19/251 (7.6)	41/776 (5.3)	14/773 (1.8)	
Death rate (per 100 patient-years)	4.49	5.57	4.15	1.39	
Hazard ratio (95 % CI)	1.25 (0.63; 2.46)		0.33 (0.18; 0.61)		1.16 (0.65; 2.06)
MMSE over time (change from baseline)					
6 months					
*n*	215	202	673	671	
Mean (SD)	–0.49 (3.41)	–0.25 (3.01)	–0.22 (2.77)	0.27 (2.63)	
Effect size (95 % CI)	0.07 (–0.12; 0.27)		0.18 (0.07; 0.29)		*p* = 0.2410
12 months					
*n*	215	202	676	672	
Mean (SD)	–1.09 (3.84)	–1.09 (3.54)	–1.09 (3.45)	–0.34 (3.16)	
Effect size (95 % CI)	0.0 (–0.19; 0.19)		0.23 (0.12; 0.33)		*p* = 1.00
18 months					
*n*	215	202	676	672	
Mean (SD)	–1.8 (3.89)	–1.86 (4.24)	–1.73 (4.03)	–0.84 (3.64)	
Effect size (95 % CI)	–0.01 (–0.21; 0.18)		0.23 (0.12; 0.34)		*p* = 0.8231
24 months					
*n*	215	202	676	672	
Mean (SD)	–2.10 (4.14)	–2.35 (4.48)	–2.15 (4.41)	–1.12 (3.87)	
Effect size (95 % CI)	–0.06 (–0.25; 0.13)		0.25 (0.14; 0.36)		*p* = 0.8832
DAD over time (change from baseline)					
12 months					
*n*	201	182	620	628	
Mean (SD)	–7.70 (18.34)	–6.58 (18.59)	–6.10 (15.38)	–3.94 (13.29)	
Effect size (95 % CI)	0.06 (–0.14; 0.26)		0.15 (0.04; 0.26)		*p* = 0.2227
24 months					
*n*	202	182	620	628	
Mean (SD)	–13.33 (20.79)	–11.78 (20.76)	–9.99 (17.31)	–7.11 (15.96)	
Effect size (95 % CI)	0.07 (–0.13; 0.28)		0.17 (0.06; 0.28)		*p* = 0.0237
New nursing home placement (change from baseline (for 12 months) or from 12 months (for 24 months))					
12 months					
*n*	100	81	252	249	
Number (%)	3 (3)	9 (11.1)	4 (1.6)	3 (1.2)	
Relative risk (95 % CI)	3.70 (1.04; 13.23)		0.76 (0.17; 3.36)		
χ^2^, *p* value	4.76, *p* = 0.0292		0.13, *p* = 0.7153		
24 months					
*n*	48	52	149	161	
Number (%)	1 (2)	4 (7.7)	5 (3.4)	1 (0.6)	
Relative risk (95 % CI)	3.70 (0.43; 31.89)		0.19 (0.02; 1.57)		
χ^2^, *p* value	1.65, *p* = 0.1985		3.05, *p* = 0.0808		
New nursing home placement at 24 months (%)	5	18.8	5	1.8	

*DAD* Disability Assessment for Dementia, *MMSE* Mini-Mental State Examination, *SD* standard deviation

*n* = the number of death cases; *J* = the number of subjects in each subgroup

TEAEs were as expected for a cholinergic agent and similar regardless of memantine use (Additional file 5: Table S5). In memantine users, serious TEAEs occurred in 17.1 % of placebo patients and 23.1 % of galantamine patients (Additional file 6: Table S6). Eighty-six percent of placebo patients and 85 % of galantamine patients with serious TEAEs were hospitalized, with death occurring in 26 % and 31 %, respectively. In memantine nonusers, TEAEs occurred in 10.4 % of placebo patients and 9.2 % of those on galantamine. Placebo patients were hospitalized at a 64 % rate, as compared with 90 % for galantamine patients, with resultant mortalities of 42 % in placebo patients and 17 % in galantamine patients. TEAEs leading to death are shown in Additional file 7: Table S7. Deaths not consequent to TEAEs are summarized in Additional file 4: Table S4.

### Outcome: MMSE

**Fig. 2:**
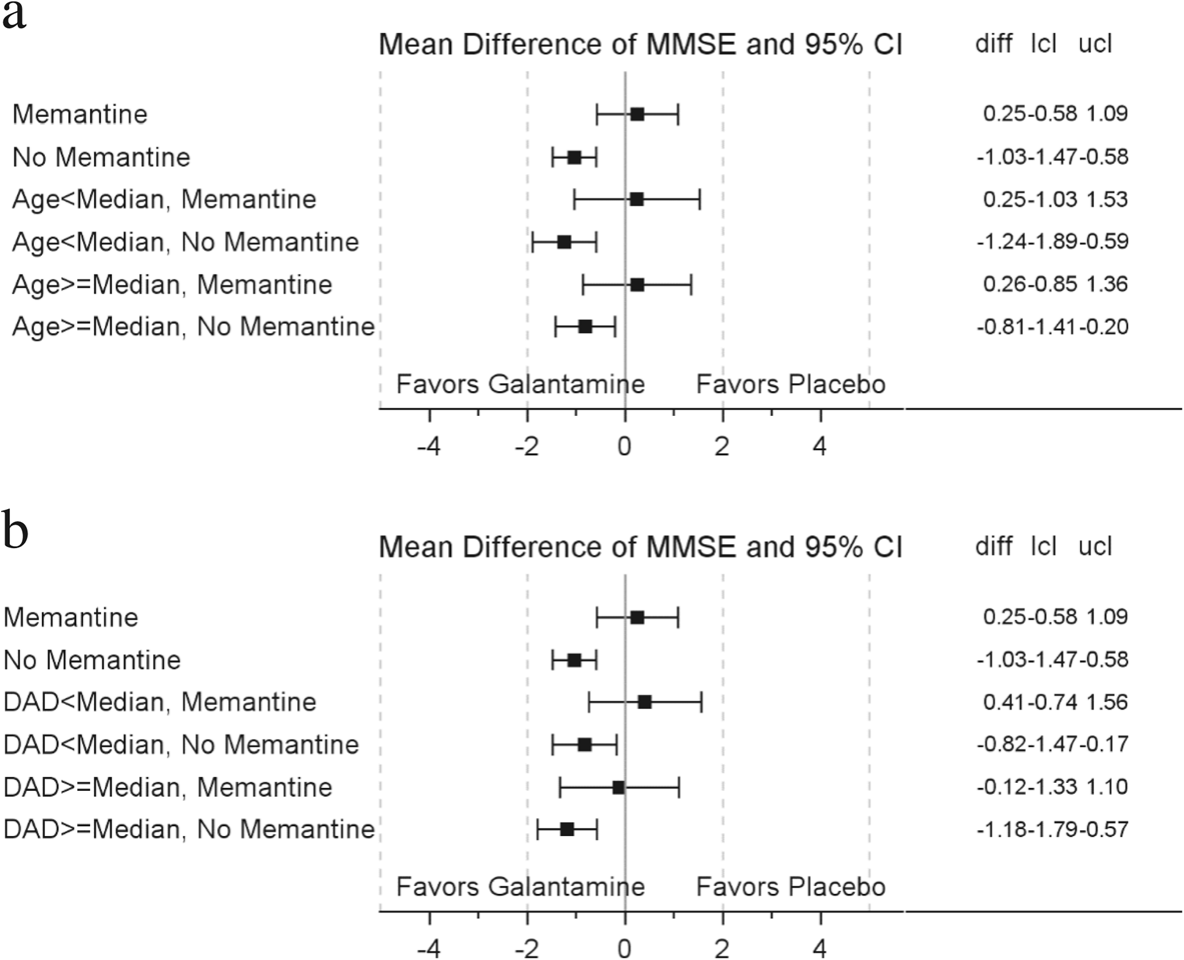
**a** Mean difference of MMSE score by median age and memantine. Two sites (049134 and 049137) excluded from the analysis due to GCP noncompliance. Median age = 74. **b** Mean difference of MMSE score by DAD score and memantine. *DAD* Disability Assessment for Dementia, *diff* difference, *MMSE* Mini-Mental State Examination, *lcl* lower confidence limit, *ucl* upper confidence limit

**Fig. 3:**
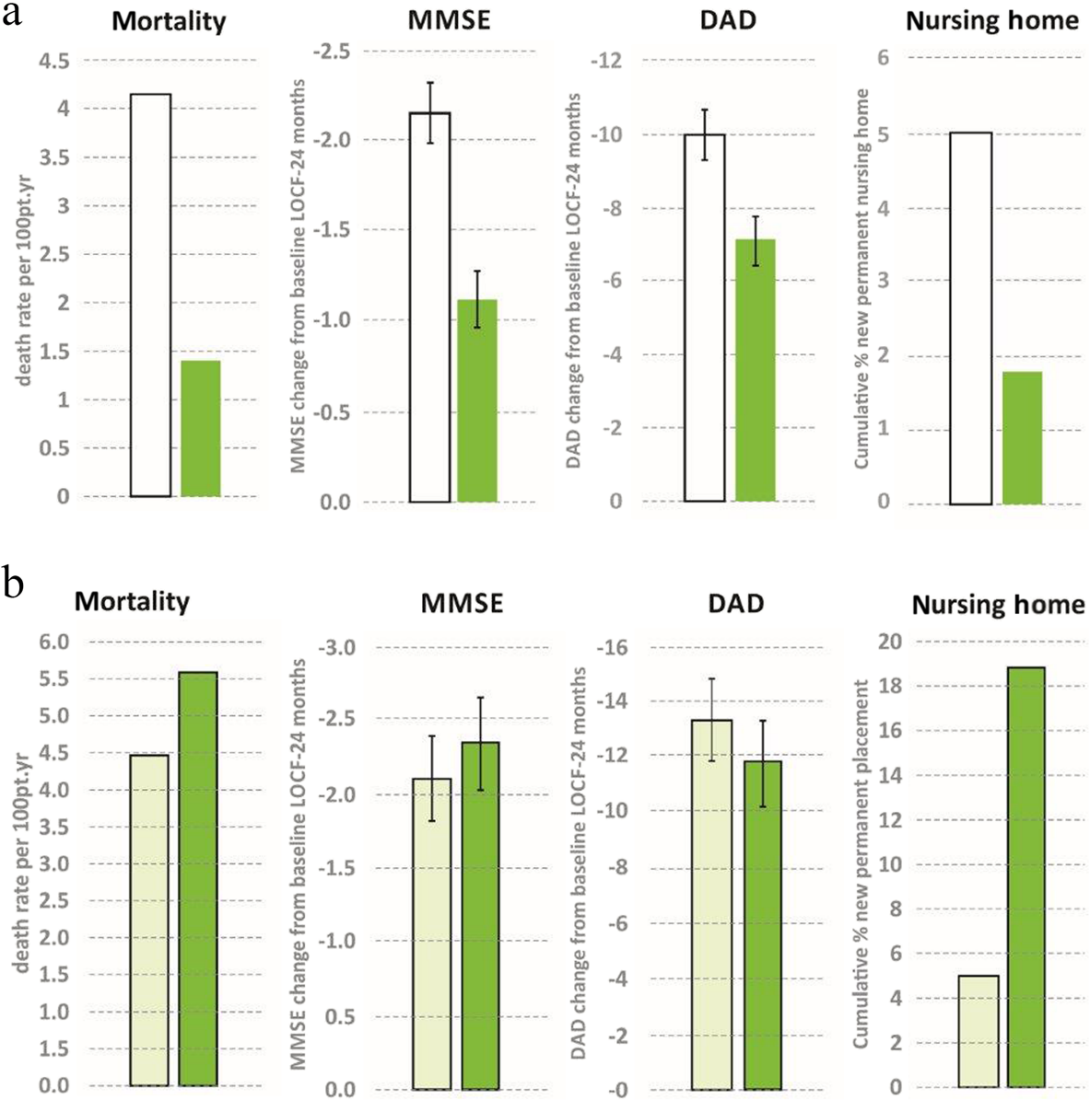
Mortality, cognitive, and functional outcomes of 24 months’ treatment with galantamine (dark green) or placebo (white or light green): **a** memantine nonusers and **b** memantine users. *DAD* Disability Assessment for Dementia, *MMSE* Mini-Mental State Examination

In memantine users, galantamine did not reduce MMSE decline at any time point (Table 2). In contrast, in memantine nonusers the galantamine group showed reduced decline in MMSE scores as compared with the placebo group at all time points, with a numerical increase in the effect size over time. The difference between memantine users and nonusers was maintained in all baseline age and DAD subgroups (Fig. 2a, b). At 2 years in memantine nonusers, scores of the galantamine group declined by –1.12 (0.15) points from baseline compared with –2.15 (0.17) points in placebo patients (Fig. 3a, b).

### Outcome: DAD

**Fig. 4:**
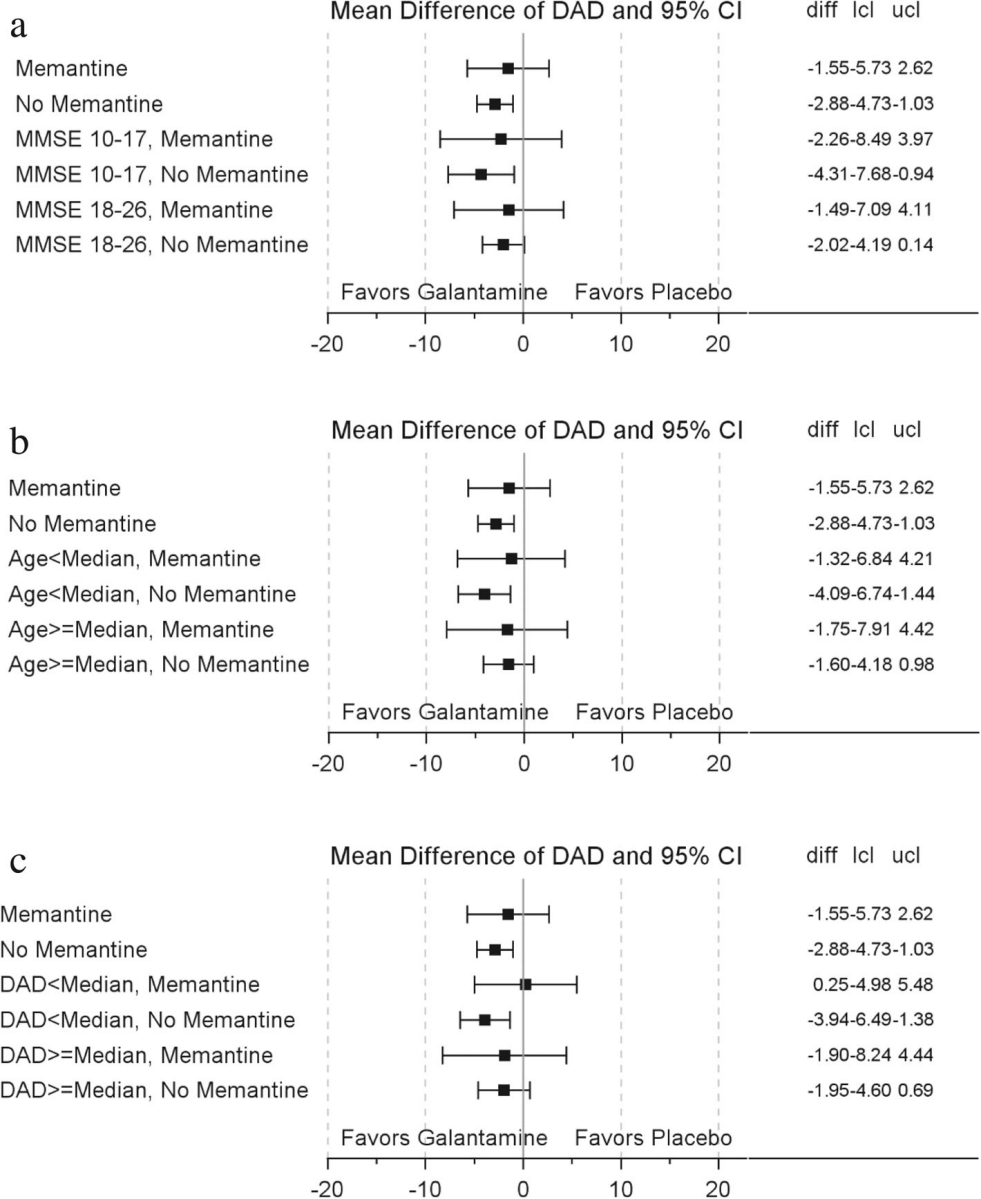
**a** Mean difference of DAD score by MMSE score and memantine. Two sites (049134 and 049137) excluded from the analysis due to GCP noncompliance. **b** Mean difference of DAD score by median age and memantine. Two sites (049134 and 049137) excluded from the analysis due to GCP noncompliance. Median DAD score = 62.16. **c** Mean difference of DAD score by DAD score and memantine. *DAD* Disability Assessment for Dementia, *diff* difference, *MMSE* Mini-Mental State Examination, *lcl* lower confidence limit, *ucl* upper confidence limit

Examination of DAD scores at month 24 demonstrated a benefit in galantamine-treated memantine nonusers, with attenuation of this benefit in the memantine user group across the range of baseline MMSE scores. A similar effect was noted in patients below the median age (Fig. 4a–c).

### Outcome: nursing home placement

In memantine users, the risk of new nursing home admission during year 1 was higher in the galantamine group than in the placebo group (3.70 (95 % CI: 1.04; 13.23), χ^2^ = 4.76, *p* = 0.03). In memantine nonusers, the risk of nursing home placement tended to be lower in galantamine-treated patients than in placebo-treated patients in year 2 (relative risk, 0.19 (95 % CI: 0.02; 1.57), χ^2^ = 3.05, *p* = 0.08). The cumulative numerical percentages of nursing home placements were 5.0 % and 18.8 % in memantine users on placebo and galantamine, respectively, and 5.0 % and 1.8 % in memantine nonusers on placebo and galantamine.

### Outcome: comparison of placebo groups

Mortality rates and MMSE scores at all time points did not differ between memantine users and nonusers randomized to placebo. Decline in the DAD was greater in users at 2 years but not at 1 year. Incident nursing home placement was equal in the two groups at 2 years.

## Discussion

Cholinesterase inhibitors are commonly prescribed for patients with mild-to-moderate AD but are often used for less than 1 year [9–11]. This short-term use may result from a commonly accepted view expressed recently in a major journal that AD responds only marginally and briefly to currently available drugs [12]. The appropriateness of less than 1 year of treatment is challenged by the first report of this randomized trial, showing that galantamine effects persist through 2 years of treatment, and underlines the importance of randomized clinical trials that extend well beyond 1 year [1].

The current report is a secondary analysis of the largest 2-year placebo-controlled, double-blind trial of a cholinesterase inhibitor in AD conducted to date. This post-hoc analysis separates patients entering the study on memantine therapy from those who were not using memantine. The data establish reductions of mortality, nursing home placement, cognitive loss, and activities of daily living (ADL) decline, persisting for 2 years, in galantamine patients not using memantine. However, the benefits of galantamine were not observed in patients taking background memantine.

The patients in this large study were close to “real-world” patients commonly seen in medical practices. They were not excluded for vascular abnormalities on CT or MRI, which are disqualifications for typical AD studies [13]. Mixed vascular and AD pathologies are the most common etiology of dementia in older persons [14]. Patients were not excluded for memantine use, which is prescribed outside the recommended MMSE range, often as monotherapy [15]. Memantine patients were slightly older, more demented and dysfunctional, and had more comorbidities than the nonusers, although the death rate between placebo memantine users and nonusers was similar, as was the observed rate of decline in MMSE in both groups. In contrast to memantine nonusers, a difference was noted in DAD decline at the end of 2 years but the percentage of patients permanently admitted to nursing homes was identical in placebo patients when memantine user and nonuser groups were compared. This secondary analysis provided the opportunity to investigate the role of baseline variables on the lack of response to galantamine in memantine users in a large, long-term study, as well as to produce a galantamine or placebo-only group of patients who provided 2 years of cholinesterase inhibitor treatment data to provide a systematic evidence base to inform clinical practice.

In memantine nonusers, galantamine use compared with placebo was associated with a decrease in mortality by 2/3, and a similar pattern in nursing home placement at 2 years. Cognitive loss was reduced by nearly half, and ADL loss by 29 %. Galantamine–placebo differences did not decrease over the course of 2 years. In contrast, memantine users with galantamine treatment did not show these benefits and had numerically more new nursing home placements than memantine users on placebo.

Perhaps the most noteworthy result in this study is the reduction in mortality seen with galantamine in the memantine nonusers. This reduction in mortality at 2 years is about twice that reported in earlier 6-month controlled studies of galantamine [1, 16]. The mortality reduction was not attributable to any organ system or cause of death. The positive effect on ADLs is corroborated by the nursing home placement data. Loss of ADLs has been an important predictor of institutionalization in several clinical studies [17, 18]. New nursing home admissions were numerically lower in galantamine-treated than in placebo-treated memantine nonusers.

Although observational studies of cholinesterase inhibitor administration have suggested mortality reduction and protection from nursing home placement [19, 20], these were not observed in a placebo-controlled, randomized trial of 3 years’ duration with another cholinesterase inhibitor [17, 21].

In contrast to the benefit of galantamine treatment of memantine nonusers, memantine users had no benefit of galantamine treatment on any measure. No other baseline variable—age, or MMSE or DAD scores—had an effect on the differences in mortality, cognition, and functional responses to galantamine between users and nonusers.

Galantamine-treated memantine users had a higher risk of nursing home admission than placebo-treated memantine users during the first year. However, baseline differences in nursing home residence and the small sample size in this subset make it difficult to interpret this finding.

It is possible that memantine users may have had some pharmacological interaction making galantamine less effective. Memantine blocks various nicotinic receptors at concentrations less than half of what it can be calculated to reach human brain tissue during treatment [22–24]. Memantine might thus be expected to act in an antagonistic manner, pharmacologically cancelling the nicotinic effects of galantamine. However, a recent study, shorter and smaller than the present one, and involving patients with milder AD, in which both memantine and galantamine were randomized, did not show an interaction in cognitive or functional outcomes [25]. Similarly, randomized memantine administration to moderate-severe AD patients receiving long-term donepezil treatment did not reduce donepezil’s efficacy, nor did it alter the rate of nursing home placement. Randomized memantine administration with galantamine or donepezil thus did not affect outcome measures [26, 27].

The main limitation of the memantine analysis is that memantine use was not randomized. Even though the effect of certain baseline characteristics was assessed, some unidentified characteristics of memantine patients, such as those accounting for the decision not to try a cholinesterase inhibitor in the large majority, could have accounted for the failure to respond to galantamine. Secondly, these patients had “probable AD” using clinical features without biomarker assessment, which probably reflects common clinical practice. The proportion of patients with other factors contributing to dementia could have varied between memantine users and nonusers. For example, the contribution of vascular factors was not assessed and could have affected the results, although galantamine has been shown to be effective in mixed dementia [28]. The advantage of the study’s limited exclusions is that it represents realistic clinical practice. Because of its size and duration, the study provides an experience which heretofore has not been available.

AD is a disease with a life expectancy of about 8 years from diagnosis with the onset of dementia [29]. The studies which inform clinical decision-making are usually 6 months in duration, and occasionally 1 year. Randomized, placebo-controlled, long-term studies are few, expensive, difficult, and ethically challenging at the current time.

## Conclusion

This study demonstrated that patients who were prescribed memantine before and during the study did not benefit from the addition of galantamine, and may have had increased institutionalization. Caution is thus advised regarding galantamine treatment in this population. These results may not extend to the reverse situation; that is, addition of memantine to patients receiving galantamine, or to simultaneous administration.

The outcomes in memantine nonusers suggest that the commonly accepted impression that the effects of cholinesterase inhibitors are minimal and time limited may not generalize to all members of this drug class. The benefits of galantamine on mortality, cognition, function, and placement were all maintained throughout 2 years’ treatment. While a placebo-controlled study of cholinesterase inhibitors is unlikely to be performed, a randomized, blinded comparison may be able to challenge conventional wisdom and offer extended benefits from inexpensive, currently available drugs.

## Additional files

Additional file 1 Table S1. Medical history by concomitant use/nonuse of memantine. (DOCX 13 kb)
Additional file 2: Table S2. Prior medications. (DOCX 17 kb)
Additional file 3: Table S3. Mortality by subgroup. (DOCX 14 kb)
Additional file 4: Table S4. Causes of death by concomitant use/nonuse of memantine. (DOCX 19 kb)
Additional file 5: Table S5. TEAEs by memantine use and randomization. (DOCX 14 kb)
Additional file 6: Table S6. Serious TEAEs. (DOCX 15 kb)
Additional file 7: Table S7. Serious TEAEs leading to death. (DOCX 14 kb)
Additional file 8: Investigators and institutional review boards/independent ethics committees. (XLSX 24 kb)

## Abbreviations

AD: Alzheimer’s disease
ADL: Activities of daily living
CI: Confidence interval
CT: Computed tomography
DAD: Disability Assessment in Dementia
HR: Hazard ratio
ITT: Intention to treat
LOCF: Last observation carried forward
MMSE: Mini-Mental State Examination
MRI: Magnetic resonance imaging
NMDA: *N*-Methyl-D-aspartate
