# Supplementary material for: Effect of concomitant use of memantine on mortality and efficacy outcomes of galantamine-treated patients with Alzheimer’s disease: post-hoc analysis of a randomized placebo-controlled study
Source: Alzheimers Res Ther. 2016 Nov 15;8:47. doi: 10.1186/s13195-016-0214-x (PMC5111338; doi:10.1186/s13195-016-0214-x)
Supplement: Additional file 1 Table S1. — Medical history by concomitant use/nonuse of memantine. (DOCX 13 kb) [file 13195_2016_214_MOESM1_ESM.docx]

**Additional file 1. Table S1: Medical history by concomitant use/nonuse of memantine**

|  | **Memantine** | | **No memantine** | |
| --- | --- | --- | --- | --- |
|  | **Placebo (n=245)**  **n (%)** | **Galantamine (n=251)**  **n (%)** | **Placebo (n=776)**  **n (%)** | **Galantamine (n=773)**  **n (%)** |
| **Total no of patients with condition^a^** | 225 (91.8) | 228 (90.8) | 688 (88.7) | 693 (89.7) |
| Allergic/immunologic | 9 (3.7) | 13 (5.2) | 19 (2.4) | 21 (2.7) |
| Cardiovascular | 179 (73.1) | 179 (71.3) | 497 (64) | 511 (66.1) |
| Dermatologic | 17 (6.9) | 13 (5.2) | 45 (5.8) | 35 (4.5) |
| Endocrine/metabolic | 89 (36.3) | 95 (37.8) | 208 (26.8) | 231 (29.9) |
| Eyes, ears, nose and throat | 77 (31.4) | 72 (28.7) | 262 (33.8) | 262 (33.9) |
| Gastrointestinal | 67 (27.3) | 75 (29.9) | 243 (31.3) | 240 (31) |
| Genitourinary | 69 (28.2) | 76 (30.3) | 190 (24.5) | 216 (27.9) |
| Hematopoietic/lymphatic | 14 (5.7) | 8 (3.2) | 34 (4.4) | 28 (3.6) |
| Musculoskeletal | 93 (38) | 71 (28.3) | 238 (30.7) | 262 (33.9) |
| Neurologic | 81 (33.1) | 77 (30.7) | 189 (24.4) | 193 (25) |
| Psychiatric | 57 (23.3) | 56 (22.3) | 131 (16.9) | 126 (16.3) |
| Respiratory | 32 (13.1) | 16 (6.4) | 100 (12.9) | 89 (11.5) |

^a^ Medical History and/or Currently Active Condition
