# Supplementary material for: Effect of concomitant use of memantine on mortality and efficacy outcomes of galantamine-treated patients with Alzheimer’s disease: post-hoc analysis of a randomized placebo-controlled study
Source: Alzheimers Res Ther. 2016 Nov 15;8:47. doi: 10.1186/s13195-016-0214-x (PMC5111338; doi:10.1186/s13195-016-0214-x)
Supplement: Additional file 2: Table S2. — Prior medications. (DOCX 17 kb) [file 13195_2016_214_MOESM2_ESM.docx]

**Additional file 2. Table S2: Prior medications**

|  |  | **Memantine** | | **No memantine** | |
| --- | --- | --- | --- | --- | --- |
| **Drug Class** | **Example(s)** | **Placebo**  **n (%)** | **Galantamine**  **n (%)** | **Placebo**  **n (%)** | **Galantamine**  **n (%)** |
| **Cardiovascular and cerebrovascular** |  | | | | |
| ACE inhibitors | captopril, enalapril, lisinopril | 63 (25.7) | 65 (25.9) | 183 (25.6) | 182 (23.5) |
| angiotensin II antagonists | candesartan, irbesartan, losartan | 14 (5.7) | 15 (6.0) | 23 (3.0) | 30 (3.9) |
| beta-blocking agents,  selective | atenolol, metoprolol, nebivolol | 43 (17.6) | 46 (18.3) | 97 (12.5) | 104 (13.5) |
| digitalis glycosides | digoxin, digitoxin | 5 (2.0) | 14 (5.6) | 12 (1.5) | 15 (1.9) |
| dihydropyridine derivatives | amlodipine, nifedipine | 20 (8.2) | 27 (10.8) | 66 (8.5) | 53 (6.9) |
| HMG CoA reductase  inhibitors | atorvastatin, lovastatin, simvastatin | 40 (16.3) | 42 (16.7) | 87 (11.2) | 89 (11.5) |
| organic nitrates | glyceryl trinitrate, isosorbide dinitrate | 7 (2.9) | 13 (5.2) | 23 (3.0) | 23 (3.0) |
| platelet aggregation  inhibitors | acetylsalicylic acid, clopidogrel | 63 (25.7) | 59 (23.5) | 147 (18.9) | 148 (19.1) |
| sulfonamides | furosemide, indapamide | 19 (7.8) | 23 (9.2) | 60 (7.7) | 54 (7.0) |
| vitamin K antagonists | phenprocoumon, warfarin | 10 (4.1) | 11 (4.4) | 18 (2.3) | 15 (1.9) |
| **Endocrine** |  |  |  |  |  |
| biguanides | metformin | 11 (4.5) | 12 (4.8) | 15 (1.9) | 30 (3.9) |
| sulfonamides, urea  derivatives | glibenclamide, glimepiride | 8 (3.3) | 6 (2.4) | 27 (3.5) | 26 (3.4) |
| thyroid hormone | levothyroxine | 18 (7.3) | 19 (7.6) | 34 (4.4) | 34 (4.7) |
| **Psychiatric (antianxiety)** |  |  |  |  |  |
| benzodiazepine derivatives | diazepam, lorazepam, oxazepam | 17 (6.9) | 13 (5.2) | 23 (3.0) | 28 (3.6) |
| **Psychiatric (antidepressant)** |  |  |  |  |  |
| monoamine reuptake  inhibitors | amitriptyline, doxepin, trimipramine | 6 (2.4) | 7 (2.8) | 10 (1.3) | 13 (1.7) |
| other antidepressants | mirtazapine, venlafaxine | 21 (8.6) | 14 (5.6) | 18 (2.3) | 25 (3.2) |
| selective serotonin reuptake  inhibitors | citalopram, fluoxetine, sertraline | 29 (11.8) | 25 (10.0) | 41 (5.3) | 42 (5.4) |
| **Psychiatric (antipsychotic)** |  |  |  |  |  |
| benzamides | sulpiride, tiapride | 12 (4.9) | 9 (3.6) | 12 (1.5) | 13 (1.7) |
| butyrophenones | haloperidol | 4 (1.6) | 9 (3.6) | 3 (0.4) | 6 (0.8) |
| diazepines, oxazepines,  thiazepines and oxepines | clozapine, olanzepine, quetiapine | 6 (2.4) | 5 (2.0) | 10 (1.3) | 5 (0.6) |
| other antipsychotics | risperidone | 6 (2.4) | 8 (3.2) | 11 (1.4) | 4 (0.5) |
| phenothiazines | chlorpromazine, trifluoperazine, thioridazine | 4 (1.6) | 1 (0.4) | 9 (1.2) | 12 (1.6) |
| thioxanthine derivatives | chlorprothixene | 1 (0.4) | 0 | 10 (1.3) | 4 (0.5) |
