# Supplementary material for: Effect of concomitant use of memantine on mortality and efficacy outcomes of galantamine-treated patients with Alzheimer’s disease: post-hoc analysis of a randomized placebo-controlled study
Source: Alzheimers Res Ther. 2016 Nov 15;8:47. doi: 10.1186/s13195-016-0214-x (PMC5111338; doi:10.1186/s13195-016-0214-x)
Supplement: Additional file 3: Table S3. — Mortality by subgroup. (DOCX 14 kb) [file 13195_2016_214_MOESM3_ESM.docx]

**Additional file 3. Table S3: Mortality by subgroups**

|  | **Placebo** | | **Galantamine** | | **Galantamine**  **vs. Placebo** |
| --- | --- | --- | --- | --- | --- |
|  | **n=1021 n/J^a^ (%)** | **Death rate (per 100 pt-yr)** | **n=1024 n/J (%)** | **Death rate (per 100 pt-yr)** | **Hazard ratio (95% CI)^b^** |
| **Concomitant Memantine** |  |  |  |  |  |
| use | 15/245 (6.1) | 4.49 | 19/251 (7.6) | 5.57 | 1.25 (0.63; 2.46) |
| nonuse | 41/776 (5.3) | 4.15 | 14/773 (1.8) | 1.39 | 0.33 (0.18; 0.61) |
| **Age^c^** |  |  |  |  |  |
| < median | 14/471 (3.0) | 2.21 | 11/474 (2.3) | 1.70 | 0.77 (0.35; 1.69) |
| >= median | 42/550 (7.6) | 6.10 | 22/550 (4.0) | 3.14 | 0.51 (0.31; 0.86) |
| **Baseline MMSE Score** |  |  |  |  |  |
| 10 – 17 | 34/378 (9.0) | 7.01 | 23/361 (6.4) | 4.82 | 0.68 (0.40; 1.16) |
| 18 – 26 | 22/643 (3.4) | 2.63 | 10/663 (1.5) | 1.15 | 0.44 (0.21; 0.92) |
| **Baseline DAD Score^d^** |  |  |  |  |  |
| < median | 42/516 (8.1) | 6.27 | 27/497 (5.4) | 4.03 | 0.64 (0.40; 1.04) |
| >= median | 13/497 (2.6) | 2.03 | 6/520 (1.2) | 0.90 | 0.44 (0.17; 1.16) |
| missing | 1/8 (12.5) | 9.04 | 0/7 (0.0) | 0.00 | 0.00 (0.00; 0.00) |

CI, confidence interval; DAD, Disability Assessment for Dementia; MMSE, Mini–Mental State Examination

^a^n/J = ratio of the number of death cases (n) and the number of patients in each subgroup (J)

^b^Hazard Ratio (95% CI) from the Cox proportional hazard model with treatment as a covariate.

^c^Median Age = 74

^d^Median DAD Score = 62.16
