# Supplementary material for: Effect of concomitant use of memantine on mortality and efficacy outcomes of galantamine-treated patients with Alzheimer’s disease: post-hoc analysis of a randomized placebo-controlled study
Source: Alzheimers Res Ther. 2016 Nov 15;8:47. doi: 10.1186/s13195-016-0214-x (PMC5111338; doi:10.1186/s13195-016-0214-x)
Supplement: Additional file 4: Table S4. — Causes of death by concomitant use/nonuse of memantine. (DOCX 19 kb) [file 13195_2016_214_MOESM4_ESM.docx]

**Table S4: Causes of death by concomitant use/nonuse of memantine**

|  | **Memantine** | | **No memantine** | |
| --- | --- | --- | --- | --- |
|  | **Placebo (N=245)**  **n (%)** | **Galantamine (N=251)**  **n (%)** | **Placebo (N=776)**  **n (%)** | **Galantamine (N=773)**  **n (%)** |
| **Total deaths** | 15 (6.1) | 19 (7.7) | 41 (5.3) | 14 (1.8) |
| **Deaths secondary to TEAEs 12 (4.9) 18 (7.2) 35 (4.5) 13 (1.7)** | | | | |
| **Cardiac disorders** | 5 (2) | 9 (3.6) | 13 (1.7) | 4 (0.5) |
| cardiac failure | 3 (1.2) | 6 (2.4) | 9 (1.2) | 4 (0.5) |
| myocardial infarction | 0 | 2 (0.8) | 1 (0.1) | 0 |
| arrhythmia | 1 (0.4) | 0 | 0 | 0 |
| cardiac arrest | 1 (0.4) | 0 | 3 (0.4) | 0 |
| coronary arteriosclerosis | 0 | 1 (0.4) | 0 | 0 |
| **Nervous system disorders** | 2 (0.8) | 3 (1.2) | 9 (1.2) | 4 (0.5) |
| dementia Alzheimer type | 1 (0.4) | 2 (0.8) | 4 (0.5) | 1 (0.1) |
| stroke | 1 (0.4) | 1 (0.4) | 5 (0.6) | 2 (0.3) |
| loss of consciousness | 0 | 0 | 0 | 1 (0.1) |
| **Infections, infestations** | 3 (1.2) | 2 (0.3) | 2 (0.8) | 2 (0.3) |
| pneumonia | 2 (0.8) | 2 (0.3) | 2 (0.8) | 2 (0.3) |
| sepsis | 1 (0.4) | 0 | 0 | 0 |
| **Respiratory** | 0 | 2 (0.8) | 1 (0.1) | 0 |
| aspiration | 0 | 1 (0.4) | 0 | 0 |
| pulmonary embolism | 0 | 1 (0.4) | 1 (0.1) | 0 |
| **General disorders** | 2 (0.8) | 1 (0.4) | 4 (0.5) | 0 |
| sudden death | 1 (0.4) | 1 (0.4) | 3 (0.4) | 0 |
| hypothermia | 0 | 0 | 1 (0.1) | 0 |
| multi-organ failure | 1 (0.4) | 0 | 0 | 0 |
| **Musculoskeletal** | 0 | 1 (0.4) | 0 | 0 |
| muscular weakness | 0 | 1 (0.4) | 0 | 0 |
| **Gastrointestinal** | 0 | 1 (0.4) | 0 | 0 |
| GI hemorrhage | 0 | 1 (0.4) | 0 | 0 |
| **Injury, poisoning** | 0 | 0 | 2 (0.3) | 1 (0.1) |
| carbon monoxide | 0 | 0 | 1 (0.1) | 0 |
| chemical poisoning | 0 | 0 | 0 | 1 (0.1) |
| head injury | 0 | 0 | 1 (0.1) | 0 |
| **Neoplasms** | 0 | 0 | 1 (0.1) | 0 |
| colon cancer | 0 | 0 | 1 (0.1) | 0 |
| **Renal and urinary** | 0 | 0 | 1 (0.1) | 0 |
| tubulointerstitial nephritis | 0 | 0 | 1 (0.1) | 0 |
| **Vascular disorders** | 0 | 0 | 1 (0.1) | 2 (0.3) |
| arteriosclerosis | 0 | 0 | 0 | 1 (0.1) |
| circulatory collapse | 0 | 0 | 1 (0.1) | 0 |
| malignant hypertension | 0 | 0 | 0 | 1 (0.1) |
| **Deaths not resulting from TEAEs** | **3 (1.2)** | **1 (0.4)** | **6 (0.8)** | **1 (0.1)** |
| **Cardiac disorders** | 1 (0.4) | 0 | 2 (0.3) | 0 |
| cardiac arrest | 0 | 0 | 2 (0.3) | 0 |
| myocardial ischemia | 1 (0.4) | 0 | 0 | 0 |
| **Nervous system disorders** | 0 | 1 (0.4) | 1 (0.1) | 1 (0.1) |
| stroke | 0 | 1 (0.4) | 1 (0.1) | 1 (0.1) |
| **Gastrointestinal** | 0 | 0 | 1 (0.1) | 0 |
| pancreatitis | 0 | 0 | 1 (0.1) | 0 |
| **Neoplasms** | 0 | 0 | 1 (0.1) | 0 |
| lung cancer | 0 | 0 | 1 (0.1) | 0 |
| **Renal and urinary** | 0 | 0 | 1 (0.1) | 0 |
| acute renal failure | 0 | 0 | 1 (0.1) | 0 |
| **Vascular** | 2 (0.8) | 0 | 0 | 0 |
| peripheral ischemia | 1 (0.4) | 0 | 0 | 0 |
| circulatory collapse | 1 (0.4) | 0 | 0 | 0 |

TEAE, Treatment emergent adverse event
