# Supplementary material for: Effect of concomitant use of memantine on mortality and efficacy outcomes of galantamine-treated patients with Alzheimer’s disease: post-hoc analysis of a randomized placebo-controlled study
Source: Alzheimers Res Ther. 2016 Nov 15;8:47. doi: 10.1186/s13195-016-0214-x (PMC5111338; doi:10.1186/s13195-016-0214-x)
Supplement: Additional file 5: Table S5. — TEAEs by memantine use and randomization. (DOCX 14 kb) [file 13195_2016_214_MOESM5_ESM.docx]

**Additional file 5. Table S5: Treatment emergent adverse events occurring ≥ 2% by memantine use and randomization**

|  | **Memantine** | | **No memantine** | |
| --- | --- | --- | --- | --- |
|  | **Placebo (n=245)**  **n (%)** | **Galantamine (n=251)**  **n (%)** | **Placebo (n=776)**  **n (%)** | **Galantamine (n=773)**  **n (%)** |
| **Patients with TEAE** | 136 (55.5) | 170 (67.7) | 360 (46.4) | 383 (49.5) |
| **Gastrointestinal** |  |  |  |  |
| nausea | 9 (3.7) | 21 (8.4) | 15 (1.9) | 65 (8.4) |
| vomiting | 4 (1.6) | 12 (4.8) | 7 (0.9) | 27 (3.5) |
| abdominal pain upper | 4 (1.6) | 6 (2.4) | 9 (1.2) | 11 (1.4) |
| constipation | 5 (2.0) | 5 (2.0) | 10 (1.3) | 11 (1.4) |
| diarrhea | 6 (2.4) | 4 (1.6) | 23 (3.0) | 22 (2.8) |
| **Psychiatric Disorders** |  |  |  |  |
| insomnia | 6 (2.4) | 12 (4.8) | 23 (3.0) | 29 (3.8) |
| aggression | 5 (2.0) | 8 (3.2) | 11 (1.4) | 10 (1.3) |
| anxiety | 4 (1.6) | 6 (2.4) | 14 (1.8) | 16 (2.0) |
| depression | 8 (3.2) | 6 (2.4) | 9 (1.2) | 11 (1.4) |
| restlessness | 3 (1.2) | 5 (2.0) | 6 (0.8) | 7 (0.9) |
| agitation | 7 (2.9) | 4 (1.6) | 14 (1.8) | 19 (2.5) |
| **Nervous System** |  |  |  |  |
| headache | 12 (4.9) | 10 (4.0) | 46 (5.9) | 49 (6.3) |
| dizziness, postural | 4 (1.6) | 5 (2.0) | 5 (0.6) | 14 (1.8) |
| syncope (loss of consciousness) | 7 (2.8) | 5 (2.0) | 2 (0.2) | 4 (0.5) |
| **Infections** |  |  |  |  |
| pneumonia | 6 (2.4) | 9 (3.6) | 5 (0.7) | 9 (1.2) |
| urinary tract infection | 5 (2.0) | 10 (4.0) | 15 (1.9) | 19 (2.5) |
| bronchitis | 5 (2.0) | 6 (2.4) | 11 (1.4) | 12 (1.6) |
| nasopharyngitis | 5 (2.0) | 5 (2.0) | 15 (1.9) | 19 (2.5) |
| **General Disorders** |  |  |  |  |
| fatigue | 4 (1.6) | 10 (4.0) | 8 (1.0) | 13 (1.7) |
| **Cardiac Disorders** |  |  |  |  |
| cardiac failure | 4 (1.6) | 11 (4.4) | 7 (0.9) | 6 (0.8) |
| **Injury, Poisoning, Procedural Complications** |  |  |  |  |
| fall | 5 (2.0) | 4 (1.6) | 6 (0.8) | 8 (1.0) |
| **Metabolism** |  |  |  |  |
| decreased appetite | 7 (2.9) | 9 (3.6) | 9 (1.2) | 22 (2.8) |
| dehydration | 1 (0.4) | 5 (2.0) | 2 (0.3) | 3 (0.4) |
| **Ear and Labyrinth Disorders** |  |  |  |  |
| vertigo | 14 (5.7) | 13 (5.2) | 34 (4.4) | 44 (5.7) |
| **Investigations** |  |  |  |  |
| weight decreased | 5 (2.0) | 9 (3.6) | 11 (1.4) | 24 (3.1) |
| blood pressure increased | 15 (6.1) | 15 (6.0) | 26 (3.4) | 44 (5.7) |
