# Supplementary material for: Effect of concomitant use of memantine on mortality and efficacy outcomes of galantamine-treated patients with Alzheimer’s disease: post-hoc analysis of a randomized placebo-controlled study
Source: Alzheimers Res Ther. 2016 Nov 15;8:47. doi: 10.1186/s13195-016-0214-x (PMC5111338; doi:10.1186/s13195-016-0214-x)
Supplement: Additional file 6: Table S6. — Serious TEAEs. (DOCX 15 kb) [file 13195_2016_214_MOESM6_ESM.docx]

**Additional file 6. Table S6: Serious treatment emergent adverse events**

|  | **Memantine** | | **No memantine** | |
| --- | --- | --- | --- | --- |
|  | **Placebo**  **(n=245)**  **n (%)** | **Galantamine**  **(n=251)**  **n (%)** | **Placebo**  **(n=776)**  **n (%)** | **Galantamine**  **(n=773)**  **n (%)** |
| **Serious adverse events** | 42 (17.1) | 58 (23.1) | 81 (10.4) | 71 (9.2) |
| **Cardiac disorders** | 7 (2.9) | 12 (4.8) | 18 (2.3) | 11 (1.4) |
| failure, insufficiency,  arrest, collapse | 4 (1.6) | 9 (3.6) | 12 (1.6) | 7 (0.9) |
| arteriosclerosis | 0 | 1 (0.4) | 0 | 0 |
| myocardial infarction | 0 | 4 (1.6) | 3 (0.4) | 3 (0.4) |
| arrhythmia | 3 (1.2) | 3 (1.2) | 1 (0.1) | 1 (0.1) |
| hypertension including  malignant or crisis | 0 | 2 (0.8) | 2 (0.3) | 2 (0.3) |
| **Nervous system disorders** | 15 (6.1) | 11 (4.4) | 27 (3.5) | 19 (2.5) |
| dementia | 5 (2) | 3 (1.2) | 9 (1.2) | 6 (0.8) |
| stroke, hemorrhagic or  ischemic | 6 (2.4) | 1 (0.4) | 9 (1.2) | 8 (1.0) |
| **Infections** | 8 (3.3) | 11 (4.4) | 8 (1.0) | 9 (1.2) |
| pneumonia | 6 (2.4) | 5 (2.0) | 4 (0.6) | 5 (0.6) |
| sepsis | 1 (0.4) | 0 | 0 | 0 |
| **Pulmonary disorders** | 1 (0.4) | 4 (1.6) | 1 (0.1) | 6 (0.8) |
| aspiration | 0 | 1(0.4) | 0 | 0 |
| pulmonary embolism | 0 | 3 (1.2) | 1 (0.1) | 2 (0.3) |
| **Injury, poisoning** | 10 (4.1) | 11 (4.4) | 12 (1.5) | 9 (1.2) |
| fall | 1 (0.4) | 1 (0.4) | 2 (0.3) | 1 (0.1) |
| fractures | 11 (3.2) | 11 (3.2) | 8 (1.0) | 6 (0.8) |
| carbon monoxide  poisoning | 0 | 0 | 1 (0.1) | 0 |
| chemical poisoning,  toxic agent exposure | 0 | 1 (0.4) | 0 | 1 (0.1) |
| head injury or  subdural bleed | 1 (0.4) | 2 (0.8) | 2 (0.3) | 0 |
| **Musculoskeletal disorders** | 3 (1.2) | 5 (2.0) | 2 (0.3) | 2 (0.3) |
| muscular weakness | 0 | 1 (0.4) | 0 | 0 |
| **Neoplasms (benign and**  **malignant)** | 3 (1.2) | 2 (0.8) | 5 (0.6) | 1 (0.1) |
| colon cancer | 0 | 1 (0.4) | 1 (0.1) | 0 |
| **Gastrointestinal disorders** | 2 (0.8) | 7 (2.8) | 6 (0.8) | 8 (1.0) |
| upper gastrointestinal  hemorrhage | 1 (0.4) | 0 | 1 (0.1) | 1 (0.1) |
| **Metabolism and nutrition disorders** | 2 (0.8) | 4 (1.6) | 2 (0.3) | 3 (0.4) |
| diabetes, hyperglycemia | 2 (0.8) | 1 (0.4) | 4 (0.5) | 1 (0.1) |
| dehydration | 0 | 2 (0.8) | 1 (0.1) | 2 (0.3) |
| **Psychiatric disorders** | 2 (0.8) | 9 (3.6) | 4 (0.5) | 3 (0.4) |
| depression | 0 | 0 | 0 | 1 (0.1) |
| aggression | 1 (0.4) | 2 (0.8) | 0 | 1 (0.1) |
| agitation, catatonia, confusion, delirium,  disorientation | 0 | 5 (2.0) | 3 (0.4) | 0 |
| psychotic disorder | 0 | 2 (0.8) | 0 | 0 |
| suicide attempt | 0 | 1 (0.4) | 0 | 0 |
| **Vascular disorders** | 2 (0.8) | 3 (1.2) | 3 (0.4) | 4 (0.5) |
| **Renal and urinary disorders** | 1 (0.4) | 2 (0.8) | 3 (0.4) | 2 (0.3) |
| nephritis | 1 (0.4) | 0 | 1 (0.1) | 0 |
| **Eye disorders** | 0 | 0 | 0 | 4 (0.5) |
| cataract | 0 | 0 | 0 | 3 (0.4) |
| glaucoma | 0 | 0 | 0 | 2 (0.3) |
| **General disorders** | 3 (1.2) | 4 (1.6) | 6 (0.8) | 0 |
| sudden death | 1 (0.4) | 1 (0.4) | 2 (0.3) | 0 |
| death | 0 | 0 | 1 (0.1) | 0 |
| hypothermia | 1 (0.4) | 0 | 1 (0.1) | 0 |
| multi-organ failure | 1 (0.4) | 0 | 0 | 0 |
