# Supplementary material for: Effect of concomitant use of memantine on mortality and efficacy outcomes of galantamine-treated patients with Alzheimer’s disease: post-hoc analysis of a randomized placebo-controlled study
Source: Alzheimers Res Ther. 2016 Nov 15;8:47. doi: 10.1186/s13195-016-0214-x (PMC5111338; doi:10.1186/s13195-016-0214-x)
Supplement: Additional file 7: Table S7. — Serious TEAEs leading to death. (DOCX 14 kb) [file 13195_2016_214_MOESM7_ESM.docx]

**Additional file 7. Table S7: Serious treatment emergent adverse events leading to death**

|  | **Memantine** | | **No memantine** | |
| --- | --- | --- | --- | --- |
|  | **Placebo**  **(n=245)**  **n (%)** | **Galantamine**  **(n=251)**  **n (%)** | **Placebo**  **(n=776)**  **n (%)** | **Galantamine**  **(n=773)**  **n (%)** |
| **Deaths** | 6.1% | 7.6% | 5.3% | 1.8% |
| **Cardiovascular** | 5 (2.0) | 9 (3.6) | 14 (1.8) | 6 (0.8) |
| cardiac, cardiovascular,  cardiopulmonary, cardio-respiratory insufficiency, failure or arrest, circulatory collapse | 4 (1.6) | 6 (2.2) | 13 (1.7) | 4 (0.5) |
| arteriosclerosis | 0 | 1 (0.4) | 0 | 1 (0.1) |
| myocardial infarction | 0 | 2 (0.8) | 1 (0.1) | 0 |
| arrhythmia | 1 (0.4) | 0 | 0 | 0 |
| malignant hypertension | 0 | 0 | 0 | 1 (0.1) |
| **Neurologic** | 2 (0.8) | 3 (1.2) | 9 (1.2) | 4 (0.5) |
| dementia Alzheimer's type | 1 (0.4) | 2 (0.8) | 4 (0.5) | 1 (0.1) |
| stroke, hemorrhagic or ischemic | 1 (0.4) | 1 (0.4) | 5 (.6) | 2 (0.3) |
| loss of consciousness | 0 | 0 | 0 | 1 (0.1) |
| **Infection** | 3 (1.2) | 2 (0.8) | 2 (0.3) | 2 (0.3) |
| pneumonia | 2 (0.8) | 2 (0.8) | 2 (0.3) | 2 (0.3) |
| sepsis | 1 (0.4) | 0 | 0 | 0 |
| **Pulmonary** | 0 | 2 (0.8) | 1 (0.1) | 0 |
| aspiration | 0 | 1 (0.4) | 0 | 0 |
| pulmonary embolism | 0 | 1 (0.4) | 1 (0.1) | 0 |
| **Injury, poisoning** | 0 | 0 | 2 (0.3) | 1 (0.1) |
| carbon monoxide poisoning | 0 | 0 | 1 (0.1) | 0 |
| chemical poisoning | 0 | 0 | 0 | 1 (0.1) |
| head injury | 0 | 0 | 1 (0.1) | 0 |
| **General disorders** | 2 (0.8) | 1 (0.4) | 4 (0.5) | 0 |
| sudden death | 1 (0.4) | 1 (0.4) | 2 (0.3) | 0 |
| death | 0 | 0 | 1 (0.1) | 0 |
| hypothermia | 0 | 0 | 1 (0.1) | 0 |
| multi-organ failure | 1 (0.4) | 0 | 0 | 0 |
| **Other** | 0 | 1 (0.4) | 3 (0.4) | 0 |
| muscular weakness | 0 | 1 (0.4) | 0 | 0 |
| upper gastrointestinal hemorrhage | 0 | 0 | 1 (0.1) | 0 |
| colon cancer | 0 | 0 | 1 (0.1) | 0 |
| tubulointerstitial nephritis | 0 | 0 | 1 (0.1) | 0 |
